# The effect of somatic mutations in mitochondrial DNA on the survival of patients with primary brain tumors

**DOI:** 10.3325/cmj.2024.65.111

**Published:** 2024-04

**Authors:** Siti Zulaikha Nashwa Mohd Khair, Siti Muslihah Ab Radzak, Zamzuri Idris, Anani Aila Mat Zin, Wan Muhamad Amir Wan Ahmad, Abdul Aziz Mohamed Yusoff

**Affiliations:** 1 Department of Neurosciences, School of Medical Sciences, Universiti Sains Malaysia, Health Campus, Kubang Kerian, Kelantan, Malaysia; 2 Department of Pathology, School of Medical Sciences, Universiti Sains Malaysia, Health Campus, Kubang Kerian, Kelantan, Malaysia; 3 School of Dental Sciences, Universiti Sains Malaysia, Health Campus, Kubang Kerian, Kelantan, Malaysia

## Abstract

**Aim:**

To assess the presence of mitochondrial (mt) DNA somatic mutations, determine the relationship between clinicopathological characteristics and mutations, and assess the survival outcomes in Malay patients with primary brain tumors.

**Methods:**

The study enrolled 54 patients with primary brain tumors. DNA extracted from paired tissue and blood samples was subjected to Sanger sequencing to identify alterations in the entire mtDNA. The associations between clinicopathological characteristics and mutations were evaluated. Cox-regression multivariate analysis was conducted to identify factors significantly associated with survival, and Kaplan-Meier analysis was used to compare the survival of patients with and without mutations.

**Results:**

Overall, 29.6% of the patients harbored 19 somatic mutations distributed across 15 loci within the mtDNA. Notably, 36.8% of these mutations were not previously documented in MITOMAP. One newly identified mutation caused a frameshift in the *ATPase6* gene, resulting in a premature stop codon. Three mutations were classified as deleterious in the MitImpact2 database. Overall, 1097 mtDNA polymorphisms were identified across 331 different locations. Patients with mutations exhibited significantly shorter survival than patients without mutations.

**Conclusions:**

mtDNA mutations negatively affected the survival outcomes of Malaysian patients with primary brain tumors. However, studies with larger samples are needed to confirm the association between mutation burden and survival rates.

According to the National Cancer Registry of Malaysia data from 2007, brain cancer and other nervous system cancers were among the top five most frequently detected cancers in men. Brain cancer is also the second most prevalent childhood neoplastic disease, accounting for estimated 5230 diagnoses in children between 0 to 19 years of age (https://www.cancer.net/cancer-types/brain-tumor/statistics) (1-5).

Brain tumor prognosis depends on tumor pathology according to the World Health Organization classification. Individuals diagnosed with primary malignant brain tumors face an extremely poor prognosis and low survival chances (6). Despite advancements in diagnosing, prognosing, and treatment, it remains crucial to screen for highly pathogenic genetic alterations, as the recent effective treatments vary in availability.

The mitochondria serve as vital bioenergetic and biosynthetic machinery, capable of establishing complex structured networks that impact numerous cellular functions. They are consistently exposed to damage due to their proximity to the respiratory chain, which is why mitochondrial DNA (mtDNA) exhibits a 10-fold higher mutation rate than nuclear DNA. These mutations reduce the efficiency of the oxidative phosphorylation (OXPHOS) system and increase reactive oxygen species (ROS) production, the known mutation source. Consequently, these events lead to oxidative stress, damaging more mtDNA (7,8). The accumulation of damaged mtDNA causes a decline in mitochondrial function, initiating tumorigenesis and sustaining cancer progression (9). However, chemoresistance of tumor cells has complicated the effort to evaluate the association of mutations with cancer pathogenesis and progression. These mutations occur in both encoded tumor suppressor genes and oncogenes found in nuclear and mtDNA (10).

The multifaceted carcinogenesis process involves various factors that influence tumor initiation, growth, progression, and potential treatments. Notably, the interactions between the nucleus and mitochondria have become a focal point of research interest, prompting researchers to assess the effect of mtDNA alterations on cancer development and progression (11). Therefore, this study aims to identify somatic mutations across the entire mtDNA genome and examine the associations between multiple characteristics and the survival status of brain tumor patients.

## Patients and methods

### Tumor and blood specimens

Between 2017 and 2020, 54 Malays with brain tumors were recruited from Hospital Universiti Sains Malaysia. This study adhered to the principles of the Declaration of Helsinki and was approved by the Research Ethics Committee of Universiti Sains Malaysia. Written informed consent for obtaining tumor and whole blood samples was gathered from all patients before neurosurgical procedures. Patients with a history of chemotherapy or radiotherapy were not included. All patients were of Malay ethnicity, as the mutation spectrum varies across different ethnicities (12). The histopathological diagnosis was made by a consultant neuropathologist following World Health Organization standards (13), and patients with metastasis history were also excluded. All samples were stored at -80 °C without repeated freezing-thawing to preserve the DNA quality.

### DNA extraction

Total DNA was extracted from all biopsies and blood samples with the QIAamp DNA Mini Kit (Qiagen, Hilden, Germany), following the manufacturer's recommendation. The DNA quality was evaluated with the NanoDrop ND1000 spectrophotometer (Thermo Fisher Scientific Inc., Waltham, MA, USA), while DNA integrity was assessed with 1% agarose gel electrophoresis.

### Entire mtDNA amplification with polymerase chain reaction (PCR)

The entire mtDNA sequence was amplified with eleven overlapping primer sets with PCR (14). The amplification process was carried out with the SureCycler 8800 Thermal Cycler (Agilent Technologies Inc., Santa Clara, CA, USA) and the KOD-Plus-Neo PCR kit (Toyobo, Osaka, Japan). The resulting DNA fragments varied in size (982 bp to 2170 bp). PCR was performed under the following conditions: 2 min initial denaturation at 94 °C; 28 to 37 cycles of 10 s (98 °C), 30 s (58 °C), and 1 min (68 °C). The PCR products were purified using the MinElute PCR Purification Kit (Qiagen, Hilden, Germany) and sequenced.

### Sanger sequencing and somatic mutation analysis

Sanger sequencing was conducted using the Big Dye Terminator cycle sequencing kit with a 96-capillary system on an Applied Biosystems 3730 Series Genetic Analyzer (Thermo Fisher Scientific Inc.). The electropherogram results were manually analyzed with ClustalW Multiple Alignment Tool in BioEdit, version 7.0.5.3, software (Ibis BioSciences, Carlsbad, CA, USA) and BLAST from NCBI *(http://www.ncbi.nlm.nih.gov/blast*), then further compared with the revised Cambridge Reference Sequence of the human mtDNA (NC_012920) from the MITOMAP database (http://www.mitomap.org).

### Statistical analysis

Differences between the groups were assessed with the Pearson χ^2^ test. Differences in survival rate were evaluated with the Kaplan-Meier curve and the log-rank test. The Cox proportional hazards survival regression multivariate analysis was conducted to estimate the association between clinicopathological characteristics and survival rate. The level of statistical significance was set at *P* < 0.05. Data analysis was performed with IBM SPSS, version 26 (IBM Corp., Armonk, NY, USA).

## RESULTS

### Patients’ characteristics

Clinicopathological characteristics are summarized in Table 1. Patients were classified into those with glial (46.3%; 25/54) and non-glial tumors (53.7%; 29/54), with non-glial tumors being predominantly represented by meningioma cases. The sample included 30 men (55.6%), with an average age of 39.8 years (4-73 years). Thirty-four (63.0%) patients had low-grade tumors, while 20 (37.0%) had high-grade tumors. Most patients had tumors localized in the supratentorial region (75.9%). Thirty-three (61.1%) tumors measured >4 cm.

**Table 1:** Clinicopathological characteristics of patients with primary brain tumor associated with mutation status

	No. (%) of patients			
Parameter	total	with mutation (%)	without mutation (%)	P*
Total	54 (100.0)	21 (38.9)	33 (61.1)	
Sex				0.708
male	30 (55.6)	10 (33.3)	20 (66.7)	
female	24 (44.4)	11 (45.8)	13 (54.2)	
Age, years				0.128
<40	25 (46.3)	7 (28.0)	18 (72.0)	
≥40	29 (53.7)	14 (48.3)	15 (51.7)	
Histological tumor types (grade)				0.876
Glial tumors:	25 (46.3)	10 (40.0)	15 (60.0)	
schwannoma (I)	2 (3.7)	0 (0.0)	2 (100.0)	
pilocystic astrocytoma (I)	2 (3.7)	1 (50.0)	1 (50.0)	
subependymal giant cell astrocytoma (I)	2 (3.7)	2 (100.0)	0 (0.0)	
astrocytoma (II)	3 (5.6)	0 (0.0)	3 (100.0)	
ependymoma (II)	2 (3.7)	0 (0.0)	2 (100.0)	
anaplastic astrocytoma (III)	2 (3.7)	0 (0.0)	2 (100.0)	
anaplastic pleomorphic xanthoastrocytoma (III)	5 (9.3)	3 (60.0)	2 (40.0)	
glioblastoma multiforme (IV)	7 (12.9)	4 (57.1)	3 (42.9)	
Non-glial tumors:	29 (53.7)	11 (37.9)	18 (62.1)	
craniopharyngioma (I)	2 (3.7)	1 (50.0)	1 (50.0)	
meningioma (I)	15 (27.8)	5 (33.3)	10 (66.7)	
meningioma (II)	4 (7.4)	2 (50.0)	2 (50.0)	
neurocytoma (II)	2 (3.7)	1 (50.0)	1 (50.0)	
medulloblastoma (IV)	6 (11.1)	2 (33.3)	4 (66.7)	
Histological tumor grades				0.480
low grade (WHO grade I and II)	34 (63.0)	12 (35.3)	22 (64.7)	
high grade (WHO grade III and IV)	20 (37.0)	9 (45.0)	11 (55.0)	
Tumor localization				0.180
supratentorial	41 (75.9)	18 (43.9)	23 (56.1)	
infratentorial	13 (24.1)	3 (23.1)	10 (76.9)	
Tumor size				0.070
≤4cm	21 (38.9)	6 (28.6)	15 (71.4)	
>4cm	33 (61.1)	15 (45.4)	18 (54.6)	
Clinical manifestations				0.291
distinct symptoms:	28 (51.9)	9 (32.1)	19 (67.9)	
generalized:				
headache	32 (59.2)			
memory loss/ cognitive dysfunction	19 (35.2)			
seizures	17 (31.5)			
personality changes	11 (20.4)			
nausea and vomiting	23 (42.6)			
focal:				
sensory deficits	7 (12.9)			
motor deficits	21 (38.9)			
visual deficits	17 (31.5)			
language deficits/ aphasia	9 (16.7)			
dizziness, balance problems/ ataxia	20 (37.0)			
combined symptoms (generalized and focal)	26 (48.1)	12 (46.2)	14 (53.8)	

*Pearson χ^2^ test with adherence to two-sided values.

**Table 2.** Mitochondrial DNA mutations discovered in patients with primary brain tumors

### Somatic mtDNA mutations in primary brain tumor

Sequencing data analysis revealed that 29.6% (16/54) of patients harbored 19 somatic mutations at 15 different loci in mtDNA (Table 2). These mutations were distributed across the entire mtDNA genome, affecting *12S rRNA* (6.7%), *16S rRNA* (20.0%), *ND2* (13.3%), *ATPase6* (20.0%), *COIII* (6.7%), *ND4* (20.0%), and *D-loop* (13.3%). Notably, 36.8% (7/19) of these mutations (A1167G, A2874G, 2980delT, 8894delA, C8897G, 9949insG, and C16560A) were not previously documented in the MITOMAP database.

**Figure 1:**
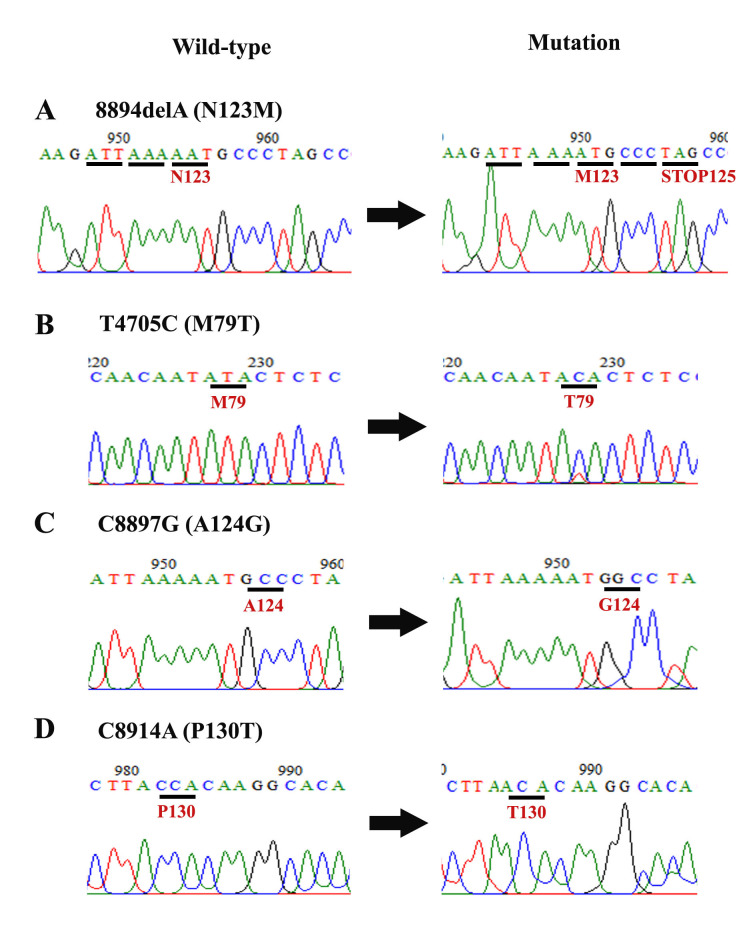
Chromatograms of wild-types and mutations detected in patients with primary brain tumors. (**A**) The deletion observed at locus 8894 of *ATPase6* led to a frame-shift mutation. Additionally, three other mutations of *ND2* and *ATPase6* were identified as having deleterious status according to MitImpact2: (**B**) T4705C, (**C**) C8897G, and (**D**) C8914A. Black lines represent the codon, while amino acid changes are in red.

An A deletion nucleotide at locus 8894 in the *ATPase6* gene was determined after aligning multiple sequencing results, compared with control samples in the BioEdit software. The deletion led to a frame-shift mutation causing early termination of protein translation (L125STOP), following the N123M protein alteration (Figure 1A). Additionally, three other mutations: T4705C, C8897G, and C8914A, found in *ND2* and *ATPase6*, were identified as deleterious in the MitImpact2 database (Figures 1B to 1D).

Various previously documented mutations were observed in the *ND4* coding-region (C10900T, C11215T, and C11665T) and one in the *D-loop* region (A16247G). Of all the recorded mutations, 57.9% (11/19) were homoplasmic and 31.6% (6/19) led to non-synonymous protein alterations.

### Polymorphic variations across the entire mtDNA in primary brain tumors

Throughout the entire mtDNA, 1097 polymorphisms were identified across 331 distinct loci, encompassing both coding and control regions (Table 3). The alterations were considered polymorphisms if they were detected in both tissue samples and corresponding blood samples. mtDNA polymorphisms were predominantly located in the *D-loop* region, spanning 90 (27.2%) different loci, with 406 (37.0%) total observed cases. A common *D-loop* polymorphism was found in 51 patients (12.6% of 406 cases), across nucleotide positions 303 to 315 (D310). This polymorphism, previously reported as mitochondrial microsatellite instability, showed variations from C7TC5 to C7TC6, C8TC6, and C9TC6 (9). Other frequently detected polymorphisms were T16519C (10.6%), T489C (6.9%), and T16362C (5.4%). Detailed information on all the polymorphisms discovered in this study can be found in Supplemental Table 1.(Supplementary Table 1)

**Table 3:** Mitochondrial genes exhibited polymorphic variants identified at various loci in patients with primary brain tumors^†^

Region of loci	Total loci (%)	Total cases (%)
*D-loop*	90 (27.2)	406 (37.0)
*12S rRNA*	11 (3.3)	65 (5.9)
*16S rRNA*	9 (2.7)	20 (1.8)
*tRNA-Leu1*	1 (0.3)	1 (0.1)
*ND1*	17 (5.1)	41 (3.7)
*tRNA-Gln*	1 (0.3)	1 (0.1)
*ND2*	20 (6.0)	36 (3.3)
*tRNA-Trp*	1 (0.3)	1 (0.1)
Non-coding (np*.5580-5586)	1 (0.3)	1 (0.1)
*tRNA-Cys*	1 (0.3)	1 (0.1)
*tRNA-Tyr*	1 (0.3)	1 (0.1)
Non-coding (np.5892-5903)	1 (0.3)	2 (0.2)
*COXI*	25 (7.6)	42 (3.8)
*COXII*	10 (3.0)	28 (2.6)
Non-coding (np.8270-8294)	1 (0.3)	14 (1.3)
*tRNA-Lys*	1 (0.3)	1 (0.1)
*ATPase8*	3 (0.9)	4 (0.4)
*ATPase6*	14 (4.2)	48 (4.4)
*COXIII*	14 (4.2)	33 (3.0)
*tRNA-Gly*	2 (0.6)	16 (1.5)
*ND3*	6 (1.8)	43 (3.9)
*ND4L*	4 (1.2)	12 (1.1)
*ND4*	11 (3.3)	15 (1.4)
*tRNA-His*	1 (0.3)	1 (0.1)
*tRNA-Ser2*	2 (0.6)	3 (0.3)
*tRNA-Leu2*	1 (0.3)	1 (0.1)
*ND5*	33 (10.0)	102 (9.3)
*ND6*	16 (4.8)	24 (2.2)
*tRNA-Glu*	2 (0.6)	3 (0.3)
*CytB*	30 (9.1)	130 (11.9)
*tRNA-Thr*	1 (0.3)	1 (0.1)
Total	331 (100)	1097 (100)

*np - nucleotide position.

†The listed mitochondrial genes display the total variants detected across various loci found in both tumor and peripheral blood samples.

### Association of clinicopathological characteristics with somatic mtDNA mutations

The mutation status was tested for associations with clinicopathological characteristics, including sex, age, histological tumor types, grades, localization, and tumor size (Table 1). Clinical presentations were categorized as distinct symptoms (generalized or focal symptoms) and combined symptoms (both symptoms experienced concurrently). A higher mutation frequency was observed in patients with combined symptoms, patients with a low-grade tumor, patients aged ≥40, patients with tumors in the supratentorial brain region, and patients with tumor size exceeding 4 cm, but none of these associations reached statistical difference.

### Clinicopathological characteristics related to a three-year survival

**Figure 2:**
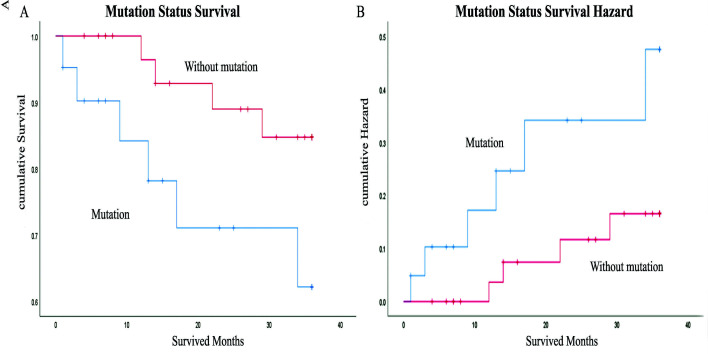
Kaplan-Meier survival analysis and hazard plots were used to assess the overall survival of patients with primary brain tumors. The three-year survival associated with mutations (*P* = 0.035) (**A**), with its hazard plot (**B**). Blue curves: mutation; Red curves: no mutation.

Cox regression analysis was used to assess the effect of multiple factors on survival time. The only variable that significantly affected survival was mutation status (*P* = 0.035). Consequently, the Kaplan-Meier analysis was performed to compare the survival of patients with and without mutations. In the analysis, patients with mutations (mean: 28.143; 95% CI 22.389-33.898) exhibited shorter survival times, approximately 28 weeks, than those without mutations (mean: 33.519; 95% CI 31.110-35.928), who survived up to 33 weeks. Kaplan-Meier curves demonstrated a lower likelihood of survival for patients with mutations, while an increased hazard implied a higher risk of mortality (Figure 2).

## DISCUSSION

This study identified 19 somatic mutations distributed across the complete mtDNA genome at 15 distinct loci. Seven of these mutations (A1167G, A2874G, 2980delT, 8894delA, C8897G, 9949insG, and C16560A) were not documented in the MITOMAP database, which makes them novel mutations.

Better screening methods increase the possibilities of detecting cancer. However, in Malaysia, minimal awareness of cancer symptoms is making early detection difficult. In fact, the lifestyle and habits of individuals, such as the use of tobacco and alcohol, and betel quid chewing contribute to mtDNA alterations and consequent tumorigenesis (29,30).

Alterations in mtDNA disrupt mitochondrial homeostasis and normal activities in response to modifications in ROS levels, calcium-dependent pathways, and energetic stress. This disruption induces dysfunction in the retrograde signaling pathway, which is initiated when mitochondria undergo dysfunction or lose their membrane potential. This extends the cell’s replicative lifespan through the involvement of multiple signal transduction proteins. In summary, alterations in mtDNA modify protein translation, leading to a reduced OXPHOS capacity and thus causing cellular energy deprivation. These events trigger stress signals that contribute to mitochondrial biogenesis inhibition. These changes in cellular metabolic and functional states also affect the nuclear gene expression profile, in addition to modifying cell morphology and physiology, ultimately leading to tumorigenesis (11,31,32).

The greatest number of distinct mutations was observed at three specific loci (*16S rRNA*, *ATPase6*, and *ND4*). Mutations such as G2975A, A2874G, and 2980delT detected within *16S rRNA* are believed to contribute to the initiation and progression of brain tumors (33). Messenger RNA translation into mitochondrial proteins involves the participation of two ribosomal RNAs (12S rRNA and 16S rRNA) encoded within the mitochondrial genome. The *12S* and *16S rRNA* genes account for approximately 1/16 and 1/10 of the entire mitochondrial genome, respectively. In human mtDNA, 297 (31%) nucleotide substitutions have been documented in the *12S rRNA* gene, and 413 (25%) in the *16S rRNA* gene (33).

In this study, we identified the mutation G2975A in the *16S rRNA* (MT-RNR2), consistent with previous reports in colorectal cancer cell lines (15). Chihara et al proposed that alterations in the *MT-RNR1* or *MT-RNR2* genes could lead to an unstable stem structure of mitochondrial ribosomal RNAs (15). The location at 2975 is analogous to the peptidyltransferase region of *E coli’s 23S rRNA* (domain V), which is susceptible to antibiotics, thereby inhibiting bacterial protein synthesis. Consequently, a mutation at position 2975 may result in a reduced mitochondrial translation activity (15).

Three mutated loci were identified in the *ATPase6* gene (C8914A, C8897G, and 8894delA). The mutation 8894delA involved a deletion of an A nucleotide at locus 8894, causing a frameshift. This led to an early termination (L123STOP) of protein translation following the N125M protein change. This mutation is categorized as a nonsense mutation introducing a premature stop codon and is likely to have a significant detrimental effect (34). The other two mutations were missense mutations predicted to have deleterious pathogenicity, which means that they underwent purifying selection, as indicated in the MitImpact2 database (34,35).

The functional role of *ATPase6* variants in tumorigenesis remains debated. The *ATPase6* gene is part of the complex V genes responsible for mtDNA maintenance. It was suggested to be responsible for mutations in breast cancer and might alter the energy metabolism of cancer cells, thereby playing a critical role in tumorigenesis (36). Mutations occurring in this gene during carcinogenesis might impede cancer cells development and hinder apoptosis. Both *ATPase6* and *ATPase8* contribute to neoplastic transformation by altering cellular energy capacity, inducing mitochondrial oxidative stress, and influencing apoptosis (37).

Three synonymous mutations were detected in the *ND4* gene (C10900T, C11215T, and C11665T). Synonymous alterations, often referred to as “silent mutations,” have a recognized impact on protein expression, conformation, and function (38). Two hypotheses have been proposed to explain the effects of silent mutations: the transfer of unidentified mutations involved in selective dominance to replace the wild-type genome, and the mitochondrion's potential ability to regulate its own replication (39).

Additionally, we observed two missense mutations in the *ND2* gene (T4705C, converting methionine to threonine, and T5401C, converting valine to alanine). The T4705C mutation is a non-conservative amino acid substitution in Complex I subunit, which is likely to result in changes in protein shape and function. It has been observed in various conditions, including bipolar disorder, optic neuropathy with brainstem lesions, and atypical psychosis (16-18). It is also documented in the ClinVar Miner database and was previously associated with Leigh syndrome (19). Seemingly, the T5401C variant shows the opposite effect to that of C5263T, which converts alanine to valine, and is also observed in the *ND2*. This change or substitution induces mitochondrial function transformation as the Complex I gets affected by the change; thus, it increases the number of mitochondrial ROS, reduces the mitochondrial membrane potential levels, and triggers the mitochondrial permeability transition pore opening (20). Several somatic mutations identified in this study were associated with other neurological disorders, such as sporadic Creutzfeldt-Jakob disease, mitochondrial encephalomyopathy, lactic acidosis, stroke-like episodes syndrome, and Leber hereditary optic neuropathy (25-27).

In this study, two mutations in D-loop were observed at loci 16247 and 16560. Additionally, 37% of patients and 27.2% of loci showed polymorphisms specifically within the *D-loop* region. Shakhssalim et al identified a variant at the locus of 16247 in both control participants and bladder cancer patients, classifying it as a polymorphism. However, in the current study, the variant was exclusively observed in brain tumor patients; hence, it can be characterized as a mutation (28).

A noteworthy finding in this study is the prevalence of variants within the nucleotide range of 303 to 315, known as D310, in 51 patients. This region encompasses the polycytosine (poly C) mononucleotide repeat tract, displaying multiple variants such as C7TC6, C8TC6, and C9TC6. Located in the hypervariable II segment, this region has been previously documented in various cancers, including colorectal cancer, brain tumor, gastric cancer, acute myeloid leukemia, and thyroid cancer, as a manifestation of mitochondrial microsatellite instability (16,40-43). The highly polymorphic D310 alteration is proposed as a potential early premalignant cancer marker due to its significant occurrence in the early stages of malignancy (11,44).

An additional variant, T16519C, identified in the mitochondrial *D-loop*, appeared predominantly as a polymorphism in 43 patients. This variant has been consistently reported in several studies related to brain tumors (45-48), which makes it a promising candidate for a brain tumor biomarker. *D-loop* region has a pivotal role in regulating the components of replication and transcription, crucial for maintaining mitochondrial biogenesis (11,49), and any modifications in this region may impede these processes, thereby contributing to tumorigenesis.

In our study, no significant associations were found between somatic mutations and the clinicopathological parameters. Similar results have been obtained in several studies across multiple tumor types (50,51). In our sample, more patients with mutations had low-grade tumors, tumor localized in the supratentorial part, and tumor sizes larger than 4 cm. These findings align with Larman et al’s conclusion that in the early stages of oncogenesis, pathogenic mtDNA mutations confer selective advantage, and have the capacity to disable mitochondrial function, thereby initiating tumorigenesis (50,52).

Meanwhile, survival outcomes of brain tumor patients were significantly associated with mutation status. Similarly, patients with oral cavity squamous cell carcinoma, who had pathogenic somatic mtDNA mutations had significantly poorer survival rate compared with those without mutations (51). Interestingly, another study with the same type of cancer presented contradictory results (8). Two other studies indicated that somatic mtDNA mutations were linked to a shorter survival rate in patients with pulmonary adenocarcinoma and pancreatic ductal adenocarcinoma (53,54).

In conclusion, our comprehensive screening of the entire mitochondrial genome revealed that 29.6% of primary brain tumor patients harbored somatic mtDNA mutations, with several of these mutations considered potentially harmful. These mutations may disrupt the normal mitochondrial functions, especially OXPHOS, thus contributing to tumorigenesis. Beyond somatic mutations, certain significant mtDNA polymorphisms were reported in several other brain tumor studies (45-48) and may serve as potential biomarkers for initial brain tumor screening in individuals.

Additionally, the observed significant correlations between mutation burden, patients’ survival, and clinicopathological parameters can offer valuable insights into the onset and brain tumor progression. However, a potential limitation of our study is the sample size. Larger-scale future studies are warranted to validate the current results and ensure the generalizability of these results. Further exploration into the detailed mechanisms linking mtDNA mutations and mitochondrial dysfunction in cancer progression could enhance cancer therapeutics development.

**Table:** 

Somatic mutation	Gene	Cases, n = 54 (%)	Transition/transversion	Homo/hetero	Protein changes	Syn/ Nonsyn/FS	Phenotype	Pathogenicity predictor (MitImpact2)^†^	References
**A1167G**	*12S rRNA*	1/54 (1.9)	transition	homo	NC	NA	novel	NA	present study
**A2874G**	*16S rRNA*	1/54 (1.9)	transition	hetero	NC	NA	novel	NA	present study
**G2975A**	*16S rRNA*	1/54 (1.9)	transition	hetero	NC	NA	colorectal cancer	NA	(15)
**2980delT**	*16S rRNA*	1/54 (1.9)	NA	homo	NC	NA	novel	NA	present study
**T4705C**	*ND2*	1/54 (1.9)	transition	hetero	M79T	non-syn	bipolar disorder	deleterious	(16)
							optic neuropathy and brainstem lesions		(17)
							atypical psychosis		(18)
							Leigh syndrome (benign)		rs1603219572 (19)^‡^
**T5401C**	*ND2*	1/54 (1.9)	transition	homo	V311A	non-syn	coronary heart disease	neutral	(20)
**8894delA**	*ATPase6*	1/54 (1.9)	NA	homo	N123M	FS	novel	NA	present study
**C8897G**	*ATPase6*	1/54 (1.9)	transversion	hetero	A124G	non-syn	novel	deleterious	present study
**C8914A**	*ATPase6*	1/54 (1.9)	transversion	homo	P130T	non-syn	thyroid carcinoma	deleterious	(21)
**9949insG**	*COIII*	1/54 (1.9)	NA	homo	V248G	FS	novel	NA	present study
**C10900T**	*ND4*	2/54 (3.7)	transition	hetero	N47N	Syn	diabetes	NA	(22)
**C11215T**	*ND4*	4/54 (7.4)	transition	homo	Y152Y	Syn	atypical psychosis	NA	(18)
							coronary heart disease		(20)
							cholestatic liver disease		(23)
							metabolic syndrome		(24)
**C11665T**	*ND4*	1/54 (1.9)	transition	homo	L302L	Syn	thyroid carcinoma	NA	(21)
							sporadic Creutzfeldt-Jakob disease		(25)
							MELAS syndrome		(26)
							LHON		(27)
**A16247G**	*D-Loop*	1/54 (1.9)	transition	homo	NC	NA	bladder cancer	NA	(28)
**C16560A**	*D-Loop*	1/54 (1.9)	transversion	hetero	NC	NA	novel	NA	present study

*Abbreviations: FS - frameshift; Homo - homoplasmic; Hetero - heteroplasmic; NA - not applicable; NC - non-coding; Nonsyn - non-synonymous; Syn - synonymous; del - deletion; ins - insertion. The details regarding detected mutations in this study encompass both novel alterations and previously reported changes.

†MitImpact2 (*http://mitimpact.css-mendel.it/)* (accessed on 18 May 2023) is a pre-computed pathogenicity predictions collection that analyzed missense mutations/variations that cause nonsynonymous substitutions in human mitochondrial protein-coding genes.

‡ClinVar Miner (*http://clinvarminer.genetics.utah.edu/)* is a database containing curated variant interpretation hosted by the National Center for Biotechnology Information (accessed on 18 May 2023) (19).
